# Efficacy and safety of short-term chemotherapy for patients with spinal tuberculosis undergoing surgery in Chinese population: a meta-analysis

**DOI:** 10.1186/s13018-021-02375-9

**Published:** 2021-03-29

**Authors:** Lu Lin, Zhenyong Ke, Si Cheng

**Affiliations:** Department of Orthopedic Surgery, The Second Affiliated Hospital of Chongqing Medical University, Chongqing, 400010 China

**Keywords:** Spinal tuberculosis, Chemotherapy, Short-course, Efficacy and safety, Meta-analysis

## Abstract

**Objective:**

The aim of this meta-analysis was to systematically evaluate the clinical efficacy and safety of short-course chemotherapy (≤ 6 months) compared with the standard therapy (9–18 months) for patients with spinal tuberculosis (TB) undergoing surgery in Chinese population.

**Methods:**

In this meta-analysis, we searched electronic databases in the Cochrane Library, PubMed, Embase, China National Knowledge Infrastructure (CNKI), Chinese Science and Technology Periodical Database (VIP), and Wanfang data to determine the equivalence of short-course therapy (group A) and standard therapy (group B) for the drug therapy of TB in Chinese population up to December 24, 2019. Weighted mean difference (WMD), odds risk (OR), and their 95% confidence interval (CI) were calculated. All analyses of relevant outcome indicators were managed by using the Review Manager (RevMan) 5.2 software.

**Results:**

This meta-analysis included six trials published involving 851 patients (group A, 397; group B, 454) with spinal TB. Results showed there were no significant differences between group A and group B in clinical cure rate (OR = 0.61; 95% CI 0.19–2.00, *p* > 0.05), change of erythrocyte sedimentation rate (ESR) (WMD = − 0.75; 95% CI − 3.33 to 1.83; *p* > 0.05) and bone graft fusion rate (OR = 2.32; 95% CI 0.36–14.81, *p* > 0.05). Meanwhile, there were fewer side effects (OR = 0.37; 95% CI 0.24–0.58, *p* < 0.05) in group A compared with group B.

**Conclusions:**

The results of this meta-analysis showed that for patients with spinal TB undergoing surgery in Chinese population, short-course chemotherapy could be equivalent to the standard chemotherapy in terms of efficacy and have less side effects than the latter.

**Supplementary Information:**

The online version contains supplementary material available at 10.1186/s13018-021-02375-9.

## Introduction

Spinal tuberculosis (TB) is the most common form of extrapulmonary tuberculosis which is first reported by Pott in 1782 [1, 2]. With the rapid growth of the global population, the incidence of drug-resistant TB and the number of spinal TB patients has shown an upward trend [3, 4]. Spinal TB is labeled as “a disease of poverty” due to its higher incidence of lower-income households and poorly developed countries [5]. Usually, the symptoms are back pain, neurologic deficit or non-specific complaints such as weight loss or fever [6]. The surgical treatment of spinal TB has developed rapidly with the improvement on theory and technology in spine surgery. Surgical intervention could be performed to debride TB infection lesions, alleviate the symptoms of nerve compression, correct kyphosis, rebuild spinal stability, and help patients start daily activities as soon as possible [7]. It is well known that anti-tuberculous chemotherapy plays an important role in the treatment of spinal TB [8]. The standard chemotherapy of 9–18 months course has achieved satisfactory effectiveness in the past several decades [8, 9]. But there are still many shortcomings about standard chemotherapy, such as a long course of treatment, the high incidence of patient non-compliance, the toxic side effects, and difficulties in clinical administration. Incomplete and inadequate treatment is considered to most important factor leading to secondary drug resistance development [10].

Due to a series of problems caused by long-term chemotherapy, it is reported that shortening the chemotherapy course might be more effective and safer. Guidelines of the American Thoracic Society recommended 6–9 months course for bone, joint, and spinal TB [11]. The British Medical Research Council trials reported that the treatment of 6 months course could get a good effect on pulmonary TB patients comparing with 8 months course [12]. It was thought that there were no differences between pulmonary and extrapulmonary disease in the most commonly prescribed regimen of sensitive TB [3]. A study with 10-year follow-up period concluded that short-course chemotherapy (≤ 6 months) was also effective in the treatment of spinal TB [13]. However, patients aging less than 15 years with an initial angle of kyphosis of more than 30 degrees were excluded in this research. Wang also confirmed the effectiveness of short-course chemotherapy (≤ 6 months) on patients of spinal TB undergoing surgical debridement [14]. China has the second highest burden of the number of TB patients in the world [15]. Thus, we perform a meta-analysis of published clinical controlled studies of the short-course chemotherapy (group A) and the standard chemotherapy (group B) for patients with spinal TB undergoing surgery in Chinese population. This study aims to further understand the efficacy and safety of short-course chemotherapy regimen for better clinical application.

## Materials and methods

This meta-analysis was conducted according to the Preferred Reporting Items for Systematic Review and Meta-analysis (PRISMA) guidelines [16]. The protocol was registered in PROSPERO (CRD42020171508).

### Search strategy

We searched in the Cochrane Library, PubMed, Embase, China National Knowledge Infrastructure (CNKI), Chinese Science and Technology Periodical Database (VIP), and Wanfang data from the inception dates to December 24, 2019. The search terms included the keywords of “spinal tuberculosis,” “short-course,” “chemotherapy,” and “study.” Relevant journals and references from retrieved articles were also searched manually. The search time was set from the time each database was established to December 2019. There were no language restrictions for publication.

### Selection of research studies

For inclusion in this meta-analysis, trials were required to meet the following eligibility criteria: (1) all published reports with a controlled clinical study, prospective study or retrospective study design, randomized controlled trials (RCTs) were given priority; (2) Chinese patients suffered from Spinal TB and received surgical treatment; (3) the study compared the clinical efficacies of short-course therapy and standard therapy; and (4) at least one of the following outcomes was reported, including the clinical cure rate, bone graft fusion rate, correction of the Cobb angle, change of erythrocyte sedimentation rate (ESR), change of C-reactive protein (CRP), change of motor function scores, change of sensory function scores and side effects.

Exclusion criteria were (1) duplicate reports of earlier trials; (2) the animal experiment, case report, review, or comment; and (3) full texts were unavailable.

### Quality assessment and data extraction

Two reviewers (LL and SC) independently identified the literatures by reading the title and abstract. The full texts of relevant studies were screened to identify the finally eligible ones. Disagreements between the two reviewers were resolved by discussion and consensus. The senior author (ZK) was also consulted. The following information was extracted in this study, which included (1) basic information on the study, including research title, first author, publication time, and country; (2) baseline of the study subject characteristics, including design, sample size, age, and gender; and (3) intervention measures.

For RCTs, the Revised Jadad scale [17] was used to evaluate the quality. The Revised Jadad scale included the random sequence production (2 points), allocation concealment (2 points), appropriateness of blinding (2 points), and description of dropouts and withdrawals (1 point). The total scores were 7 points, 4–7 points meant high quality, and 0–3 points meant poor quality. Meanwhile, we used the Newcastle-Ottawa Quality Assessment Scale (NOQAS) [18] as the tool to evaluate the quality assessment of the non-RCTs. The NOQAS consisted of 9 points in 3 major categories: selection of the study population (4 points), comparability between groups (2 points), and measurement of exposure factors (3 points). When a study ≥ 6 points, it was divided into high-quality.

### Statistical analysis

We managed the data using the Review Manager (RevMan) 5.2 software (The Cochrane Collaboration, Oxford, United Kingdom). We expressed results for dichotomous outcomes as odds risk (OR) and continuous outcomes as weighted mean difference (WMD) with 95% confidence intervals (CIs). The heterogeneity of treatment effects between studies was investigated using Cochran’s *Q* test and the *I*^2^ statistic. When there were no significant differences in heterogeneity test between studies (*P*> 0.05, *I*^2^< 50%), analysis used fixed effects model. Otherwise, the random effects model was used. If there was statistical heterogeneity between the results of the study, we further analyzed the source of the heterogeneity. After excluding the effect of obvious clinical heterogeneity, we used subgroup analysis or sensitivity analysis to resolve the heterogeneity. There were significant differences between the two groups with *p* < 0.05. We also used a funnel plot for the presence of publication bias if there were more than 10 studies included.

## Results

### Study selection

**Fig. 1:**
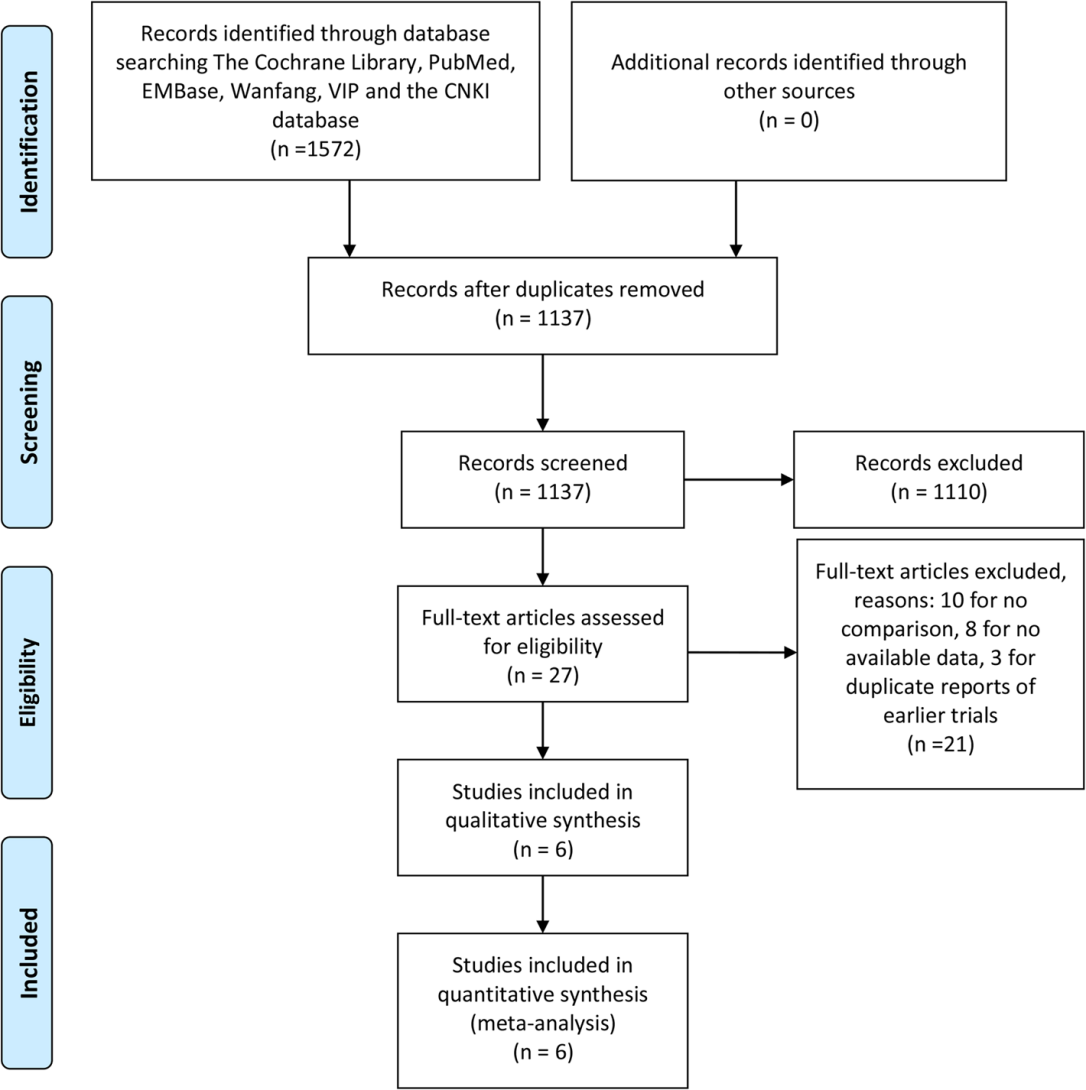
PRISMA flow diagram

Initially, we searched 1572 articles (The Cochrane Library (*n*=11), PubMed (*n*=224), Embase (*n*=304), CNKI (*n*=322), VIP (*n*=99), and Wanfang data (*n*=612) potentially eligible trials. 1408 trials were excluded for the following reasons: duplicate reports, lack of comparison, and high dropout rates. Finally, 6 articles [14, 19–23] were included in the meta-analysis. The PRISMA Flow Diagram was shown in Fig. 1 and the PRISMA checklist was detailed in Table S1 (Supplementary Material). One article [19] included three groups of 6-month course, 9-month course, and 18-month course chemotherapy, so we combined the two groups of 9- or 18-month course for better comparison.

### Study characteristics

There were 6 articles [14, 19–23] including 3 RCTs, 1 prospective study, and 2 retrospective studies. All articles were conducted in China, which included 4 articles [20–23] written in Chinese and 2 articles [14, 19] were written in English. Quality assessment showed that the 3 RCTs included 2 [19, 21] high-quality studies with scores ≥ 4 and one low-quality study [20] with scores < 4. The 3 retrospective studies were high-quality studies with scores ≥ 6. The basic characteristics of these studies were summarized in Table 1.

**Table 1:** Characteristics of studies included in the meta-analysis

References	Year of publication	Country	Design	Mean follow-up (month)	Basic data						Treatment		QAS
					Group A			Group B			Group A	Group B	
					Sample size	Age in years (mean±SD)	Male (%)	Sample size	Age in years (mean±SD)	Male (%)			
Hong Liu et al. [20]	2017	China	RCT	36	98	40.7±4.2	51(52%)	98	40.1±4.7	50(51%)	2SHRZ/2–4HRZ	2SHRZ/7–16HRZ	3
Bo Wang et al. [21]	2015	China	RCT	NA	40	42.11±2.09	28(70%)	40	42.34±2.21	27(68%)	2SHRZ/2~4HRZ	2SHRZ/10–16HRZ	4
Yongli He et al. [22]	2015	China	RS	71	100	41.7±5.4	58(%)	91	41.3±5.8	50(55%)	2SHRZ/2–4HRZ	2SHRZ/7–16HRZ	7
Yirong Zheng et al. [23]	2014	China	RS	78.6	38	NA	NA	38	NA	NA	2SHRZ/2–4HRZ	2SHRZ/7–16HRZ	6
Upadhyay et al. [19]	1996	China	RCT	175.2	25	32.6±18.3	9(36%)	89	NA	46(52%)	3SHR/3HR	3SHR/6HR or 3H+S+ PASNa/15S+ PASNa	4
Zili Wang et al. [14]	2013	China	PS	69.1	96	37.96 ± 16.3	54(56%)	89	41.53 ± 15.76	50(56%)	2SHRZ/2–4HRZ	2SHRZ/7HRZ	8

*NA* Not available, *RCT* Randomized controlled trial, *RS* Retrospective study, *PS* Prospective study, *R* Rifampicin, *H* Isoniazid, *Z* Pyrazinamide, *S* Streptomycin, *PASNa* Sodium para-aminosalicylic acid, *QAS* Quality assessment score: the Newcastle-Ottawa Scale (NOQAS) was used for assessing the quality of retrospective study or prospective study, the Revised Jadad’s scale was used for RCT

### Meta-analysis results

#### Main outcomes

##### Clinical cure rate

**Fig. 2:**
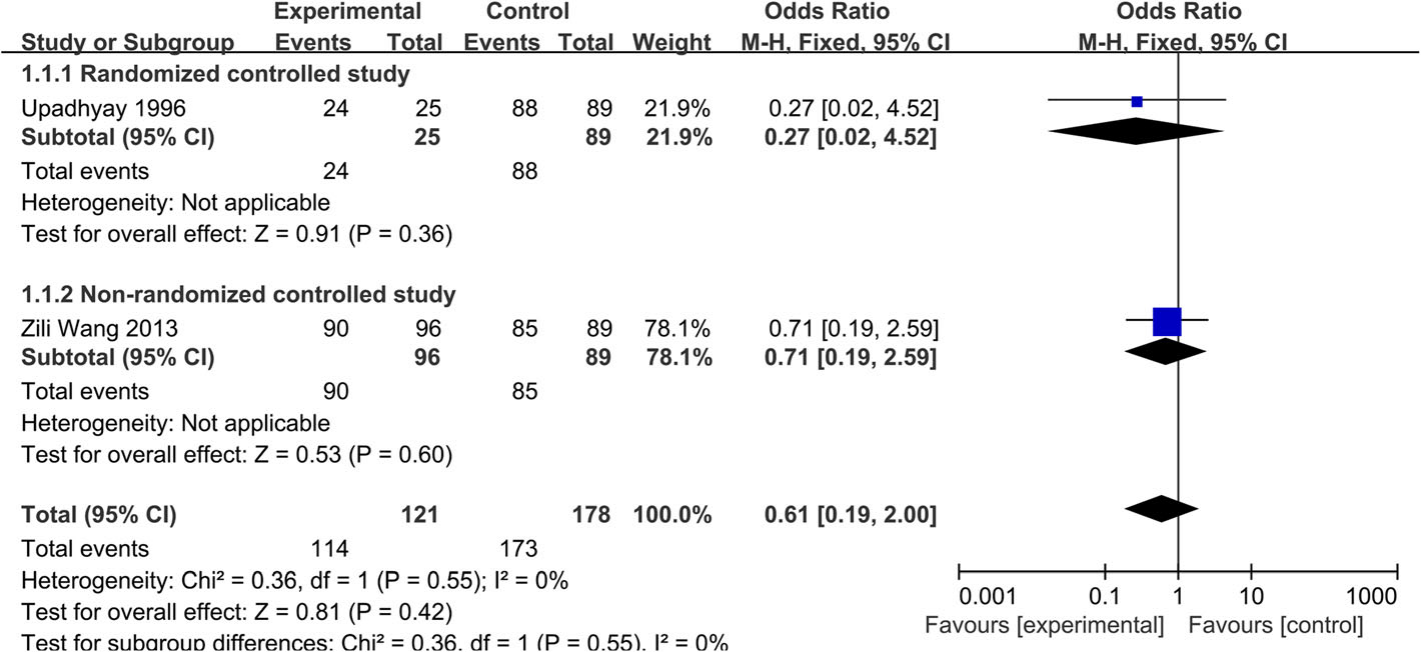
Clinical cure rate

The clinical cure was defined as disappearance of TB toxic symptoms and local pain, recovery of ability to work, and to perform activities of daily living. Two studies [14, 19] with a total of 299 patients (group A, 121; group B, 178) reported the clinical cure rate. Subgroup analysis was performed on the basis of study design. Because the heterogeneity was not significant (*P*=0.55, *I*^2^=0.0%), a fixed effects model was used to the data analysis. The combined results of data showed that there were no significant differences between group A and group B in clinical cure rate (OR=0.61; 95% CI 0.19–2.00, *p* > 0.05) (Fig. 2). It suggested that the short-course chemotherapy was no worse than the standard chemotherapy in clinical cure rate.

##### Change of ESR

**Fig. 3:**
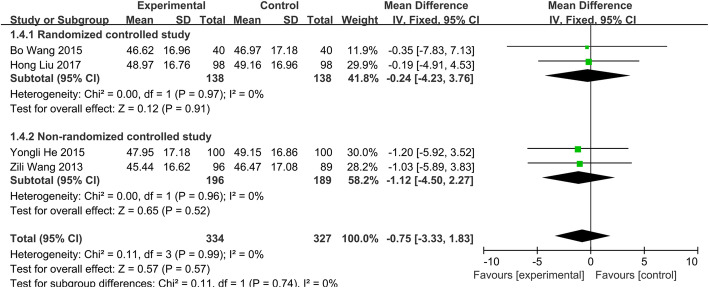
Change of ESR

Four separate studies [14, 20–22], consisting of 661 patients (group A, 334; group B, 327) were divided into two subgroups based on the different design. Testing of heterogeneity was not significant (*P*=0.99, *I*^2^=0.0%), so we used the fixed effects model to conduct the meta-analysis. No significant difference was found in the change of ESR between two groups (WMD = − 0.75; 95% CI − 3.33 to 1.83; *p* > 0.05) (Fig. 3). It meant that the short-course chemotherapy may be comparable to standard chemotherapy for the treatment of spinal TB.

##### Bone graft fusion rate

**Fig. 4:**
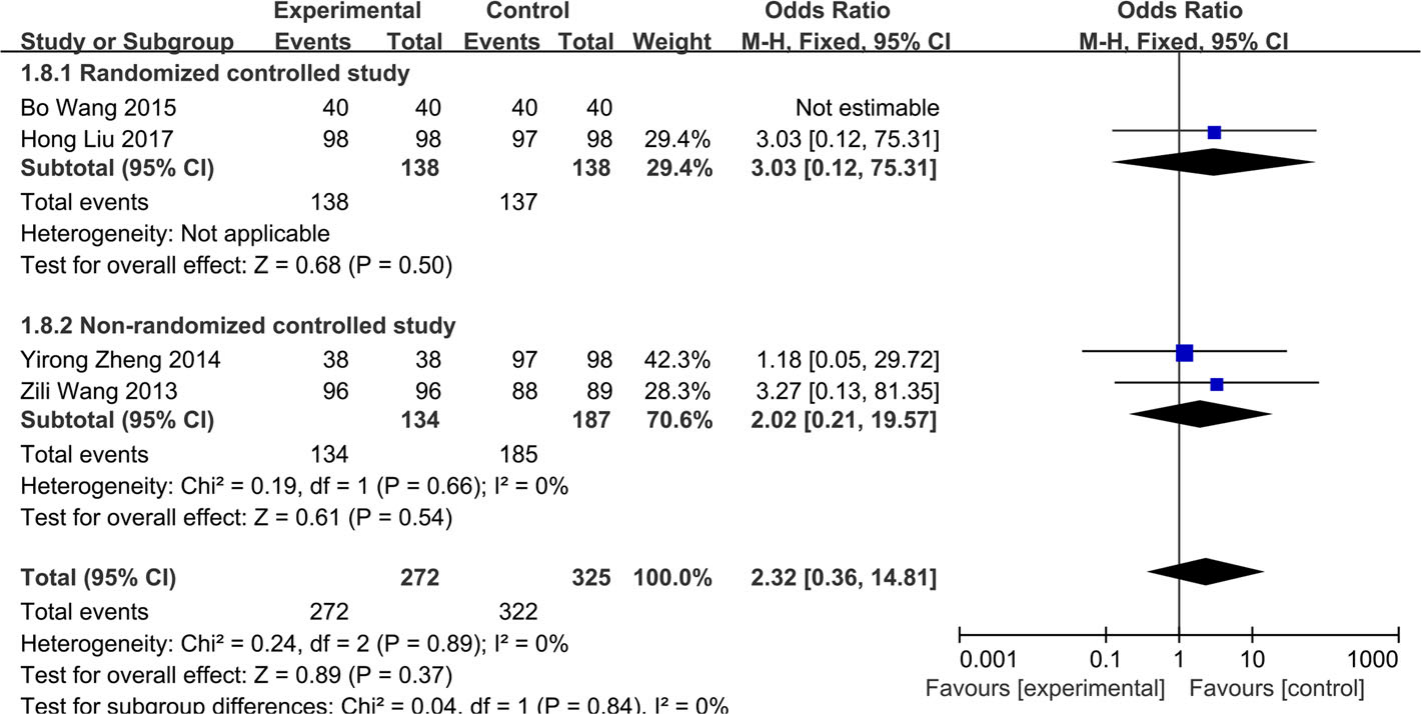
Bone graft fusion rate

Four studies [14, 20, 21, 23] reported bone graft fusion rate at the last follow-up including 597 patients (group A, 272; group B, 322). There was no statistical heterogeneity across studies (*P*=0.89, *I*^2^=0.0%), a fixed-effects model was used. There were no significant differences (OR=2.32; 95% CI 0.36–14.81, *p*> 0.05) between two groups in bone graft fusion rate (Fig. 4).

##### Side effects

**Fig. 5:**
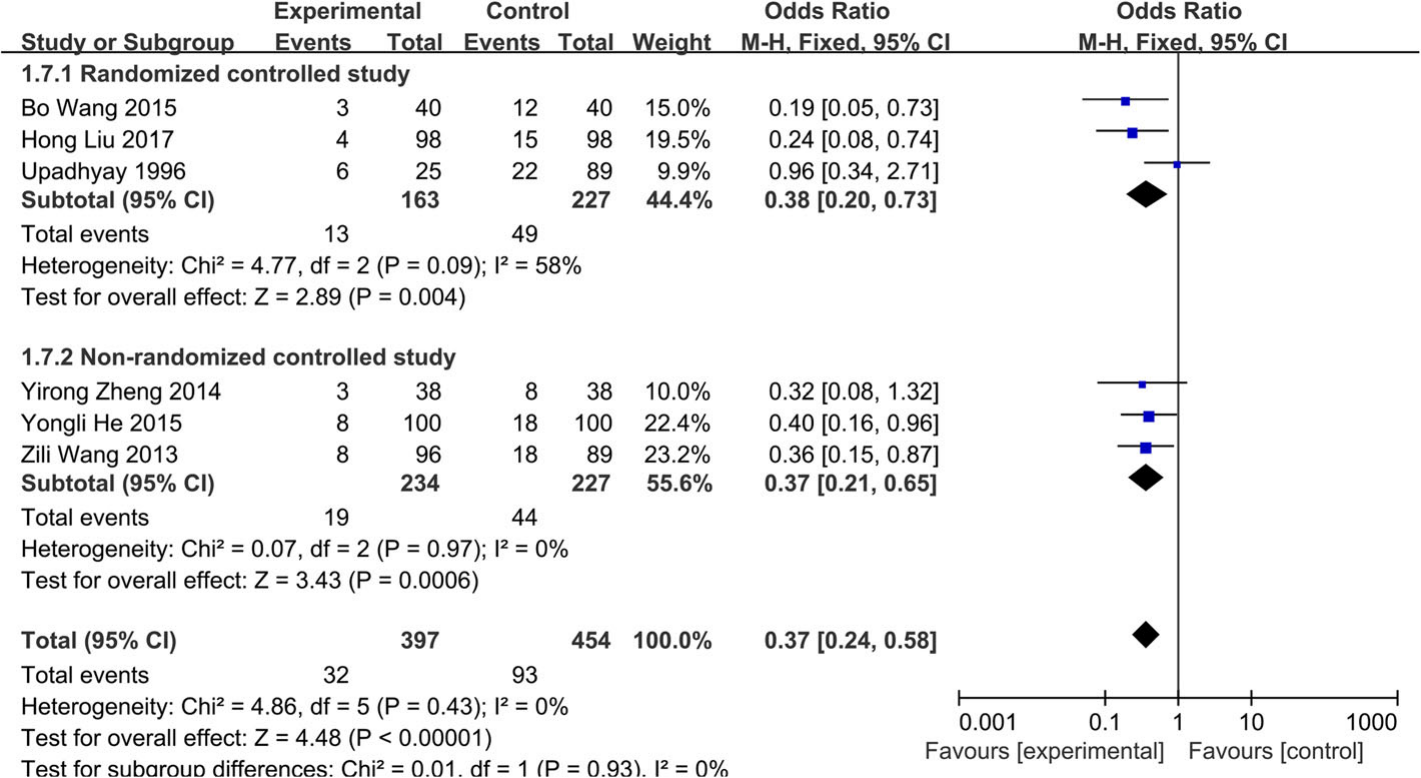
Side effects

The data of side effects was available in all six separate studies [14, 19–23], which including 851 patients (group A, 397; group B, 454). Meanwhile, these studies divided into two subgroups considering the different designs. Because of no significant statistical heterogeneity (*P*=0.43 and *I*^2^=0.0%), a fixed-effects model was used for the data analysis. The combined results showed that there were statistically significant differences between group A and group B in side effects (OR=0.37; 95% CI 0.24–0.58, *p* < 0.05) (Fig. 5). It indicated that the incidence of side effects in short-course chemotherapy was significantly lower than that in standard chemotherapy.

#### Additional outcomes

##### Correction of angle after operation

**Fig. 6:**
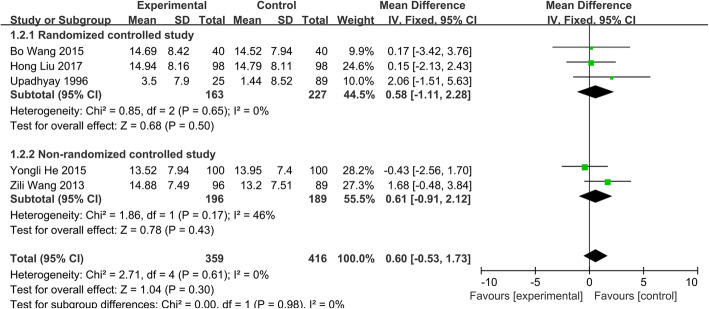
Correction of angle after the operation

The subgroup analysis was used in this data analysis based on the different designs of the study. Five studies [14, 19–22] with a total of 775 patients (group A, 359; group B, 416) reported the correction of angle after the operation. The fixed-effects model was chosen because heterogeneity was not identified (*P*=0.61, *I*^2^=0.0%). The results (Fig. 6) showed that the 95% CI range for the WMD contained zero (WMD = 0.60; 95% CI − 0.53 to 1.73; *p* > 0.05), indicating that the results did not reveal a statistically significant difference between two groups in the correction of angle after operation.

##### Change of CRP

**Fig. 7:**
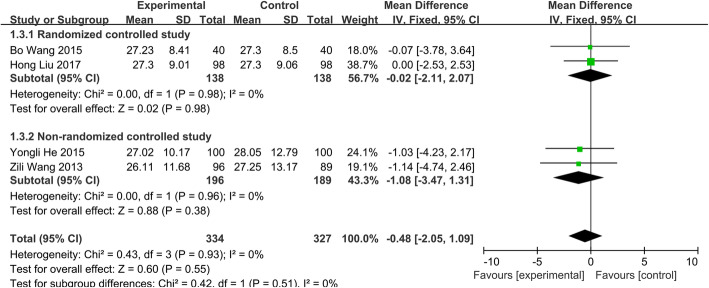
Change of CRP

The studies [14, 20–22] which divided into two subgroups, consisting of 661 patients (group A, 334; group B, 327), were included to assess the change of CRP. The fixed-effects model was used to determine the overall effects because the heterogeneity among the studies was not significant (*P*=0.93, *I*^2^=0.0%) (Fig. 7). There were no significant differences in the change of CRP between two groups (WMD = − 0.48; 95% CI − 2.05 to 1.09; *p* > 0.05).

##### Improvement of motor function score

**Fig. 8:**
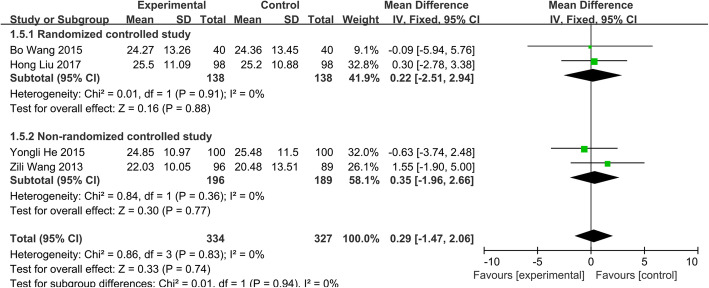
Improvement of motor function score

Four studies [14, 20–22] included 661 patients (group A, 334; group B, 327) and divided into two subgroups. We used a fixed-effects model to the Data analysis under the premise of no statistical heterogeneity (*P* > 0.83 and *I*^2^=0.0%). The pooled WMD in two groups is 0.29 (95% CI − 1.47 to 2.06, *p* > 0.05) (Fig. 8), which suggests that there were no significant differences in the improvement of motor function score comparing two chemotherapy regimens.

##### Improvement of sensory function score

**Fig. 9:**
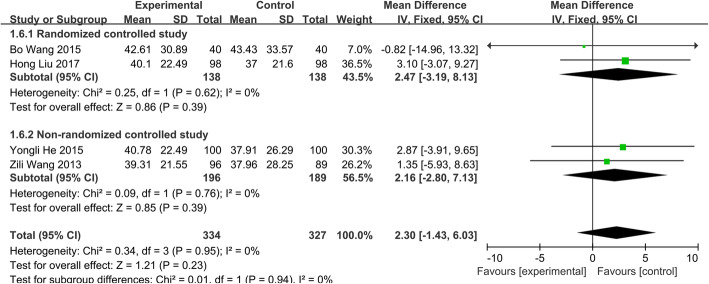
Improvement of sensory function score

The meta-analysis for improvement of sensory function score was shown in Fig. 9. We also divided four studies [14, 20–22] (group A, 334; group B, 327) into two subgroups. Meanwhile, no significant heterogeneity existed between studies (*P*=0.95, *I*^2^=0.0%), so fixed effect model was applied. There were no significant differences (WMD = 2.30; 95% CI − 1.43 to 6.03; *p*> 0.05) in improvement of sensory function score.

##### Sensitivity analysis and publication bias

For sensitivity analysis, single elimination of each study had no significant influence on the overall results. This meta-analysis included 6 studies which were less than 10, so to find out relevant bias is usually impossible. Therefore, the funnel plots were not used to evaluate the publication bias of the study results.

## Discussion

Spinal tuberculosis is classified to be a severe form of extrapulmonary tuberculosis which is accounted for 2% of all cases of TB and 15% of extrapulmonary TB cases [24]. The development of antimicrobial chemotherapy and the introduction to new antibiotics deeply revolutionized treatment for TB [25]. Anti-TB chemotherapy should follow the principles of early medication, combined medication, regularly full-course medication, and appropriate dosage [26]. The standard chemotherapy of 9-18 months course is widely used around the world. Meanwhile, we also should give attention to the management of side-effects. Rajasekaran, S pointed out that short-course chemotherapy has many inherent advantages: improved patient compliance, lower failure rates, lower cost, and lower incidence of drug resistance [27]. Directly Observed Treatment Short Course (DOTS) defined by the WHO is an effective strategy to increase cure rates in the developing world [28]. Patients could follow the drug regimen of short-course under the observation of a doctor.

Surgical intervention is necessary in cases with specific indications: no response to chemotherapy, abscess, significant kyphosis, or worse neurological status [29]. Surgeons need to choose a reasonable plan based on the individual condition of patients. The basic surgical method is debridement of TB lesions. A meta-analysis summarized that complete radical debridement had more advantages compared with incomplete radical debridement such as lower incidence of recurrence, lower rate of adverse reaction, shorten healing time, and faster recovery [30]. Three approaches to the surgical management for spinal TB included the anterior, approach, the posterior approach, and the combined approach [31]. Some scholars considered that the anterior approach had better quality in terms of debridement and bony fusion, while the posterior approach was the best mean to achieve reduction followed by stable sagittal alignment [32]. A recent systematic review showed that no justification for superiority over the three procedures [33].

It remains a controversial issue that which chemotherapy course should be selected for better clinical results and higher safety. To our knowledge, this study is the first meta-analysis designed to compare the efficacy and safety between short-course and standard chemotherapy in the treatment of spinal TB. Systematic reviews and meta-analysis are often considered the highest level of evidence. Besides, systematic reviews and meta-analysis are critical to summarize evidence which relates to healthcare interventions [16]. This meta-analysis of eight effective indicators analyzed the efficacy and safety of short-course chemotherapy versus standard chemotherapy in the treatment of spinal TB. Our results showed that short-course chemotherapy had less side effects compared with the standard group. The side effects included anaphylaxis, gastrointestinal reactions, abnormal liver, and kidney function, and auditory nerve injury. In addition, there were no differences between the two groups in the aspects about clinical cure rate, bone graft fusion rate, correction of angle after the operation, change of CRP, change of ESR, improvement of motor function score, and improvement of sensory function score. It indicated that patients might benefit from the short-course chemotherapy.

It is noted that these outcomes could not exclude the contribution of surgical treatment of TB lesions. In order to rule out this bias, all included studies underwent through debridement treatment. We found that short-course chemotherapy was also suitable for conservative treatment of spinal TB [34]. Ravinder Kumar Banga concluded that there were no significant differences in clinical, radiological, and ESR values compared 6 with 12 months of conservative therapy [35].

Drug-resistant TB was not mentioned in this meta-analysis, which was a major threat to TB control efforts. Drug resistance, especially multidrug-resistance seriously affected the prognosis of patients with spinal TB. Bacteriological confirmation and drug susceptibility testing should be sought to guide therapy [36]. The treatment course for patients with drug-resistant TB was prolonged [37]. As drug-resistant TB is always accompanied with irregular medications and side effects, this meta-analysis might provide a more simplified treatment to enhance the compliance of patients for reducing the emergence of drug-resistant TB.

Some limitations of this study should be discussed. Firstly, randomized controlled trials were the best study design to avoid biased results. Although four RCTs were included in the present article, 3 of them were high-quality. The non-RCTs were also recruited in this meta-analysis which tended to exaggerate the real results [38]. Secondly, the number of studies and patient samples was small. Many important differences might not identify some of the consequences. More high-quality randomized controlled trials were required to guide the selection. Thirdly, we were unable to conduct a comprehensive analysis incorporating other relevant clinical data. Although this meta-analysis of 6 studies included 851 patients, we considered that the observed indicators were insufficient. Finally, all recruited studies were published from China. We should interpret these results with caution, because each country had ethnic and regional differences. The inherent heterogeneity among including studies limited the strength of our conclusions. Regardless of these limitations, our results might still provide valuable information to clinicians.

## Conclusions

The results of this meta-analysis showed that for patients with spinal TB undergoing surgery in Chinese population, short-course chemotherapy could be equivalent to the standard chemotherapy in terms of efficacy and have less side effects than the latter. It still requires the clinician to make a comprehensive judgment based on the specific situation of patients.

## Supplementary Information

**Additional file 1: Table S1** PRISMA checklist

## Data Availability

All data are fully available without restriction.

## Abbreviations

TB: Tuberculosis
PRISMA: Preferred reporting items for systematic review and meta-analysis
CNKI: China national knowledge infrastructure
VIP: Chinese science and technology periodical database
RCTs: Randomized controlled trials
ESR: Erythrocyte sedimentation rate
CRP: C-reactive protein
NOQAS: Newcastle-Ottawa quality assessment scale
RevMan: Review manager
OR: Odds risk
WMD: Weighted mean difference
CIs: Confidence intervals
DOTS: Directly observed treatment short course

## References

[1] Pu X, Zhou Q, He Q, Dai F, Xu J, Zhang Z, et al. A posterior versus anterior surgical approach in combination with debridement, interbody autografting and instrumentation for thoracic and lumbar tuberculosis. Int Orthop. 2012;36(2):307–13. 10.1007/s00264-011-1329-0. 10.1007/s00264-011-1329-0 PMC3282863 21901411

[2] Gorse GJ, Pais MJ, Kusske JA, Cesario TC (1983). Tuberculous spondylitis. A report of six cases and a review of the literature. Medicine (Baltimore).

[3] Khanna K, Sabharwal S (2019). Spinal tuberculosis: a comprehensive review for the modern spine surgeon. Spine J.

[4] Yang L and Liu Z. Analysis and therapeutic schedule of the postoperative recurrence of bone tuberculosis. J Orthop Surg Res. 2013;8(Suppl 3):47. 10.1186/1749-799X-8-47 PMC3878563 24341624

[5] Dunn RN, Ben HM (2018). Spinal tuberculosis: review of current management. Bone Joint J..

[6] Qu JT, Jiang YQ, Xu GH, Tang Y, Wang ZT, Ye XJ, et al. Clinical characteristics and neurologic recovery of patients with cervical spinal tuberculosis: should conservative treatment be preferred? A retrospective follow-up study of 115 cases. World Neurosurg. 2015;83(5):700–7. 10.1016/j.wneu.2015.01.015 25681590

[7] Tang Y, Wu WJ, Yang S, Wang DG, Zhang Q, Liu X, et al. Surgical treatment of thoracolumbar spinal tuberculosis-a multicentre, retrospective, case-control study. J Orthop Surg Res. 2019;14(1):233. 10.1186/s13018-019-1252-4. 10.1186/s13018-019-1252-4 PMC6651955 31337417

[8] Guven O, Kumano K, Yalcin S, Karahan M, Tsuji S (1994). A single stage posterior approach and rigid fixation for preventing kyphosis in the treatment of spinal tuberculosis. Spine.

[9] Shi J, Yue X, Niu N, Zhao C, Qiu H, Wang Z (2017). Application of a modified thoracoabdominal approach that avoids cutting open the costal portion of diaphragm during anterior thoracolumbar spine surgery. Eur Spine J..

[10] Pawar UM, Kundnani V, Agashe V, Nene A, Nene A (2009). Multidrug-resistant tuberculosis of the spine--is it the beginning of the end? A study of twenty-five culture proven multidrug-resistant tuberculosis spine patients. Spine.

[11] Nahid P, Dorman SE, Alipanah N, Barry PM, Brozek JL, Cattamanchi A, et al. Official American thoracic society/centers for disease control and prevention/infectious diseases society of America clinical practice guidelines: treatment of drug-susceptible tuberculosis. Clin Infect Dis. 2016;63(7):e147–95. 10.1093/cid/ciw376 PMC6590850 27516382

[12] No authors listed. Short-course chemotherapy for pulmonary tuberculosis under routine programme conditions: a comparison of regimens of 28 and 36 weeks duration in Algeria. Algerian Working Group/British Medical Research Council Cooperative Study. Tubercle. 1991;72(2):88-100. 10.1016/0041-3879(91)90034-p 1949222

[13] Parthasarathy R, Sriram K, Santha T, Prabhakar R, Somasundaram PR, Sivasubramanian S (1999). Short-course chemotherapy for tuberculosis of the spine. A comparison between ambulant treatment and radical surgery - Ten-year report. J Bone Jt Surg Ser B.

[14] Wang Z, Shi J, Geng G, Qiu H (2013). Ultra-short-course chemotherapy for spinal tuberculosis: five years of observation. Eur Spine J.

[15] Glaziou P, Floyd K, Raviglione MC (2018). Global Epidemiology of Tuberculosis. Semin Respir Crit Care Med.

[16] Liberati A, Altman DG, Tetzlaff J, Mulrow C, Gøtzsche PC, Ioannidis JP, et al. The PRISMA statement for reporting systematic reviews and meta-analyses of studies that evaluate healthcare interventions: explanation and elaboration. BMJ. 2009;339 b2700. 10.1136/bmj.b2700 PMC2714672 19622552

[17] Jadad AR, Moore RA, Carroll D, Jenkinson C, Reynolds DJ, Gavaghan DJ, et al. Assessing the quality of reports of randomized clinical trials: is blinding necessary? Control Clin Trials. 1996;17(1):1–12. 10.1016/0197-2456(95)00134-4 8721797

[18] Stang A (2010). Critical evaluation of the Newcastle-Ottawa scale for the assessment of the quality of nonrandomized studies in meta-analyses. Eur J Epidemiol.

[19] Upadhyay SS, Saji MJ, Yau ACMC (1996). Duration of antituberculosis chemotherapy in conjunction with radical surgery in the management of spinal tuberculosis. Spine.

[20] Liu H (2017). Observation of long-term curative effect of complete debridement combined with ultra-short-course chemotherapy in the treatment of spinal tuberculosis. J Cervicodynia Lumbodynia.

[21] Wang B. Clinical study of spinal tuberculosis treated with surgery and ultra-short course chemotherapy. China J Chin Mater Med. 2016:2535–6.

[22] He YL, Li JC, Xiang JY, Yan PH, Tang W (2015). Long-term evaluation of spinal tuberculosis with surgery and ultra-short-course chemotherapy. Chin Foreign Med Res.

[23] Zheng YR (2014). Long-term effect of surgery combined with ultra-short-course chemotherapy in the treatment of spinal tuberculosis. Guide China Med.

[24] Chen CH, Chen YM, Lee CW, Chang YJ, Cheng CY, Hung JK (2016). Early diagnosis of spinal tuberculosis. J Formos Med Assoc.

[25] Riva MA (2014). From milk to rifampicin and back again: history of failures and successes in the treatment for tuberculosis. J Antibiot.

[26] Mukherjee JS, Rich ML, Socci AR, Joseph JK, Viru FA, Shin SS, et al. Programmes and principles in treatment of multidrug-resistant tuberculosis. Lancet. 2004;363(9407):474–81. 10.1016/S0140-6736(04)15496-2. 10.1016/S0140-6736(04)15496-2 14962530

[27] Rajasekaran S, Khandelwal G (2013). Drug therapy in spinal tuberculosis. Eur Spine J.

[28] AlSahafi AJ, Shah HB, AlSayali MM, Mandoura N, Assiri M, Almohammadi EL, et al. High non-compliance rate with anti-tuberculosis treatment: a need to shift facility-based directly observed therapy short course (DOTS) to community mobile outreach team supervision in Saudi Arabia. BMC Public Health. 2019;19(1):1168. 10.1186/s12889-019-7520-8 PMC6712871 31455324

[29] Rajeswari R, Balasubramanian R, Venkatesan P, Sivasubramanian S, Soundarapandian S, Shanmugasundaram TK, et al. Short-course chemotherapy in the treatment of Pott's paraplegia: report on five year follow-up. Int J Tuberc Lung Dis. 1997;1(2):152–8. 9441080

[30] JL N, ZX L and CX L. Comparison of clinical efficacy of complete and incomplete radical debridement for spinal tuberculosis: a Meta-analysis. China J Orthop Traumatol 2018;31(7):642-650. 10.3969/j.issn.1003-0034.2018.07.011 30103588

[31] Meena S, Mittal S, Chowdhary B (2014). Spinal tuberculosis: which is the best surgical approach?. Med Princ Pract.

[32] Varatharajah S, Charles YP, Buy X, Walter A, Steib JP (2014). Update on the surgical management of Pott's disease. Orthop Traumatol Surg Res.

[33] Bian Z, Gui Y, Feng F, Shen H, Lao L. Comparison of anterior, posterior, and anterior combined with posterior surgical treatment of thoracic and lumbar spinal tuberculosis: a systematic review. J Int Med Res. 2019:1219630379. 10.1177/0300060519830827 PMC7581984 30880540

[34] Darbyshire J (1999). Five-year assessment of controlled trials of short-course chemotherapy regimens of 6, 9 or 18 months' duration for spinal tuberculosis in patients ambulatory from the start or undergoing radical surgery. Fourteenth report of the Medical Research Council Working Party on Tuberculosis of the Spine. Int Orthop.

[35] Banga RK, Singh J, Dahuja A, Garg RS (2018). Spinal tuberculosis - directly observed treatment and short course or daily anti tubercular therapy -are we over treating?. Open Orthop J.

[36] Joint T (1998). Chemotherapy and management of tuberculosis in the United Kingdom: recommendations 1998. Joint Tuberculosis Committee of the British Thoracic Society. Thorax.

[37] Lange C, Dheda K, Chesov D, Mandalakas AM, Udwadia Z, Horsburgh CRJ (2019). Management of drug-resistant tuberculosis. Lancet (London, England).

[38] Kunz R, Oxman AD (1998). The unpredictability paradox: review of empirical comparisons of randomised and non-randomised clinical trials. BMJ.

